# Recent Advances in the Mechanisms of Cell Death and Dysfunction in Doxorubicin Cardiotoxicity

**DOI:** 10.31083/j.rcm2411336

**Published:** 2023-11-27

**Authors:** Tian-Hu Wang, Yan Ma, Shan Gao, Wei-Wei Zhang, Dong Han, Feng Cao

**Affiliations:** ^1^National Clinical Research Center for Geriatric Diseases, the Second Medical Center, Chinese PLA General Hospital, 100853 Beijing, China

**Keywords:** anthracycline, mechanism, cardiotoxicity, cell death, dysfunction

## Abstract

Despite recent advances in cancer therapy, anthracycline-based combination therapy remains the standardized first-line strategy and has been found to have effective antitumor actions. Anthracyclines are extremely cardiotoxic, which limits the use of these powerful chemotherapeutic agents. Although numerous studies have been conducted on the cardiotoxicity of anthracyclines, the precise mechanisms by which doxorubicin causes cardiomyocyte death and myocardial dysfunction remain incompletely understood. This review highlights recent updates in mechanisms and therapies involved in doxorubicin-induced cardiomyocyte death, including autophagy, ferroptosis, necroptosis, pyroptosis, and apoptosis, as well as mechanisms of cardiovascular dysfunction resulting in myocardial atrophy, defects in calcium handling, thrombosis, and cell senescence. We sought to uncover potential therapeutic approaches to manage anthracycline cardiotoxicity *via* manipulation of crucial targets involved in doxorubicin-induced cardiomyocyte death and dysfunction.

## 1. Introduction

The advent of chemotherapy drugs has greatly improved the survival rate of cancer patients. However, a large number of surviving cancer patients suffer from cardiac abnormalities induced by antitumor therapy [1]. Cardiovascular toxicity is one of the most prevalent and potentially fatal adverse effects of antitumor therapies, such as chemotherapy drugs [2]. Anthracyclines, a class of chemotherapy drugs, are routinely used to treat a variety of tumors, such as breast cancer, acute leukemia, sarcomas, Hodgkin’s and non-Hodgkin’s lymphomas, and other hematologic and solid tumors [3]. As a typical representative of anthracyclines, doxorubicin (DOX) has been extensively studied, and the incidence of DOX-induced cardiotoxicity (DOXIC) increases with the cumulative dose [4, 5]. DOXIC may be a continuous process that begins with a decline in the function of cardiomyocytes exemplified by decreased ${\mathrm{Ca}}^{2+}$ loading capacity and contractile dysfunction, gradually leading to dysfunction. Cumulative cellular dysfunction and persistent DOX injury trigger cell death when the adaptive processes that respond to DOX fail [6, 7]. Unlike the violent process of cell death, the process of dysfunction is gradual and progressive. Dexrazoxane (DXZ) is the only drug approved by the US Food and Drug Administration to reduce the cardiotoxicity of DOX. However, it still has some shortcomings: it can aggravate the bone marrow suppression caused by chemotherapy drugs, affect the antitumor effect of DOX, and may lead to secondary malignant tumors after long-term use [8, 9]. Besides, some clinically available drugs including β blockers, angiotensin converting enzyme inhibitors, angiotensin receptor antagonists, and statins have exhibited certain benefits in delaying the progression of heart failure induced by DOX [10, 11, 12]. However, these medications are not curative and there is still an urgent need to explore new strategies targeting the pathological mechanisms of DOXIC to develop more therapeutic strategies to prevent it in clinical practice. Understanding the recent advances in mechanisms of anthracycline cardiotoxicity is pertinent to developing more effective therapeutics to prevent or alleviate the cardiotoxic effects of anthracyclines. This review summarizes the mechanisms and implications of cardiomyocyte death and dysfunction induced by DOXIC and proposes novel and promising therapeutic interventions to manage DOXIC.

## 2. Regulated Cell Death

**Fig. 1.**
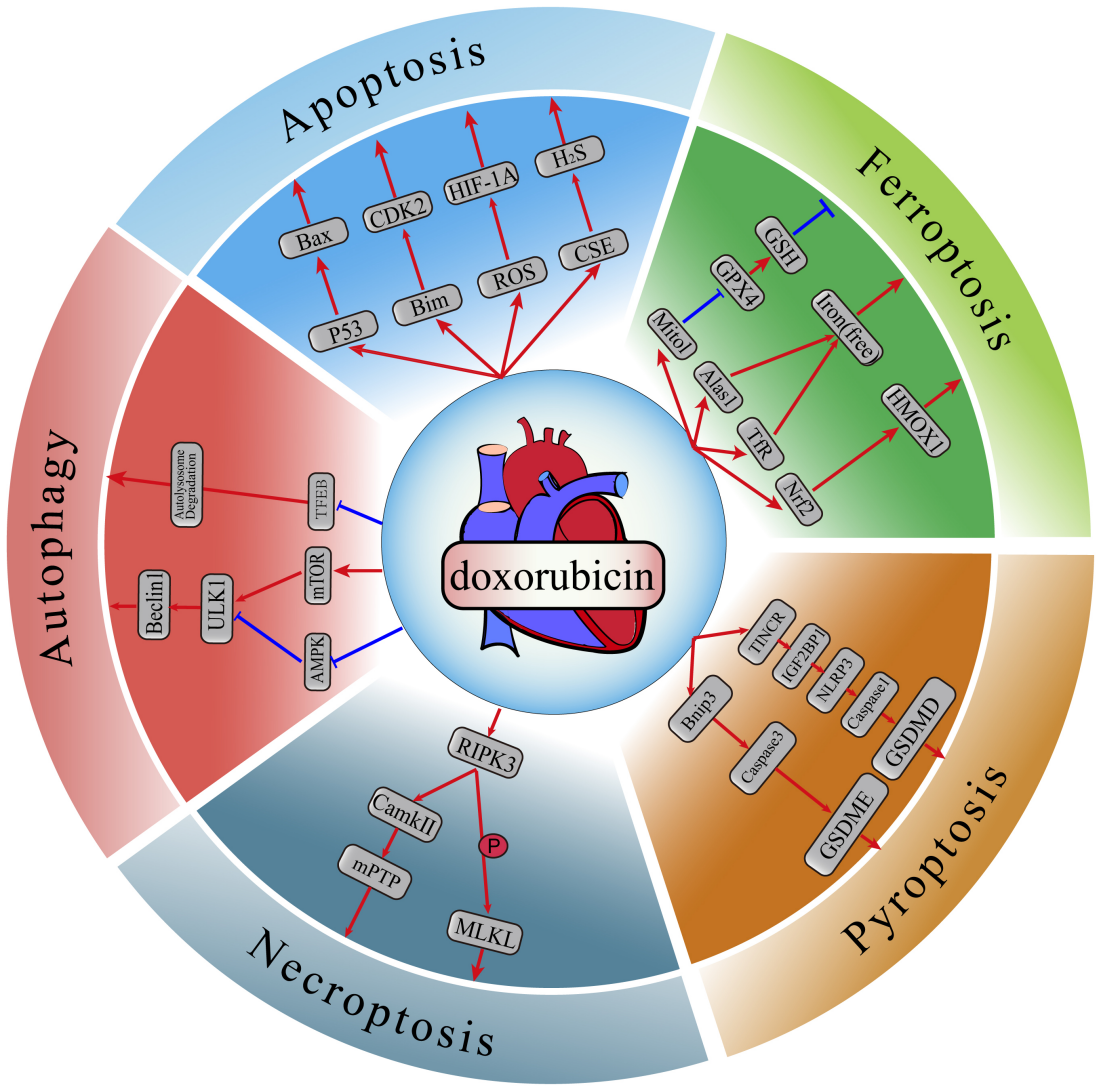
**Proposed mechanisms involved in DOX-induced cardiomyocyte regulated cell death**. Dox, doxorubicin; Alas1, aminolevulinate synthase 1; Bax, Bcl-2-associated X protein; Bnip3, Bcl-2/adenovirus E1B interacting protein 3; CamkII, calmodulin kinase II; CDK2, cyclin-dependent kinase 2; CSE, cysteine gamma-lyase; GPX4, glutathione peroxidase 4; GSDMD, gasdermin D; GSDME, gasdermin E; HMOX1, heme oxygenase-1; Mitol, E3 ubiquitin-protein ligase; MLKL, mixed lineage kinase domain like pseudokinase; mTOR, mammalian target of rapamycin; NLRP3, nod-like receptor protein 3; TFEB, transcription factor EB; TINCR, terminal differentiation-induced non-coding RNA; GSH, glutathione; TfR, transferrin receptor; Nrf2, nuclear factor erythroid 2-related factor 2; Caspase 1, cysteinyl aspartate specific proteinase 1; IGF2BP1, insulin like growth factor 2 mRNA binding protein 1; RIPK3, receptor-interacting serine-threonine kinase 3; mPTP, mitochondrial permeability transition pore; Beclin1, Bcl-2-interacting protein 1; ULK1, unc-51 like autophagy activating kinase 1; AMPK, adenosine $5^{\prime }$-monophosphate (AMP)-activated protein kinase; HIF-1A, hypoxia inducible factor 1 alpha; p53, tumor protein p53; Bim, Bcl-2 interacting mediator of cell death; ROS, reactive oxygen species. Arrows indicate activation; bar-headed lines indicate inhibition.

Regulated cell death (RCD) is implicated in general processes such as organogenesis and tissue remodeling, removal of redundant structures or cells, and regulation of cell numbers [13]. RCD can also be triggered by pathological stress when the adaptive processes that respond to stress fail. Five high-profile forms of RCD, including apoptosis, autophagy, necroptosis, ferroptosis, and pyroptosis, have been implicated in the pathogenesis of DOXIC [14]. In this review, we discuss the classical mechanisms of RCD and the specific pathways of cell death induced by DOX, to determine potential therapeutic targets to minimize the detrimental effects of DOX (Fig. 1).

## 3. Apoptosis

The most extensively studied type of cell death related to DOXIC is cell apoptosis, which is morphologically manifested as cell atrophy, increased cytoplasmic density, loss of mitochondrial membrane potential and permeability variation. As a result of these actions, complete apoptotic bodies are formed, which are absorbed and degraded by neighboring cells [15]. According to various mechanisms, apoptosis is divided into intrinsic apoptosis and extrinsic apoptosis. Intrinsic pathways, also known as mitochondrial or Bcl-2 (B-cell lymphoma 2)-mediated pathways, are activated by BH3 proteins (Bim, BMF, BID, PUMA, BIK, HRK, BAD, and NOXA) to initiate apoptosis in the face of intracellular stress caused by various physical and chemical factors [16, 17]. Downstream apoptotic factors released by mitochondria, such as cytochrome c and Smac (second mitochondria-derived activator of caspase) protein, also known as DIABLO, activate Bcl-2-associated X protein (Bax) which facilitates caspase cascade activation, which eventually results in protein cleavage and cell death [18]. In extrinsic apoptosis, the combination and activation of members of the tumor necrosis factor (TNF) receptor superfamily, the Fas/Fas ligand (FasL) system, and members of the death receptor (DR) family (DR3, DR4, and DR5) with corresponding ligands, results in the intracellular lethal signaling complex, which in turn activates caspase-8 and its effectors [19].

DNA damage in DOX-treated cardiomyocytes is thought to be the primary effect of DOXIC. Apoptosis is a secondary response to DNA damage. Topoisomerase-II is reported to be the direct target of DOX, which results in DNA double-strand breaks and opens endogenous apoptotic pathways [20]. Bcl-2 antagonist killer 1 (Bak) and Bax, members of the Bcl-2 family, oligomerize in response to DNA damage, creating a pore in the mitochondrial outer membrane that causes the release of cytochrome C and the activation of caspases, resulting in apoptosis [21]. The insulin-like growth factor 1 (IGF1) signaling pathway is a well-recognized cell survival signal and exogenous IGF1 is capable of rescuing cardiomyocytes from apoptosis triggered by DOX [22]. Endogenous hydrogen sulfide ($H_{2}$S) has recently been found to be cytoprotective in mitigating DOX-induced cardiomyocyte apoptosis. DOX significantly down-regulates the production of endogenous $H_{2}$S by inhibiting the activity of $H_{2}$S endogenous synthetase cystathionine γ-lyase (CSE), and endogenous $H_{2}$S persulfidates caspase-3 at Cysteine 163, inhibiting its activity and thus inhibiting myocardial cell apoptosis [23]. The mitochondrial outer membrane (OMM) is home to the mitochondrial GTPase fusion protein, known as mitofusin 2 (MFN2), which is crucial for the fusion and division of mitochondria. MFN2 expression in cardiomyocytes is down-regulated by DOX, while restoring MFN2 expression can reverse DOX-induced mitochondrial fission, reactive oxygen species (ROS) overproduction and apoptosis [24]. DOX can also induce apoptosis in cardiomyocytes *via* extrinsic mechanisms. DOX is reported to induce apoptosis in cardiomyocytes in a manner dependent on activation of the DR including Fas, TNF receptor 1 (TNF-R1), DR4, and DR5 [25, 26].

## 4. Autophagy

As a key mechanism of intracellular degradation, autophagy, also named type II cell death, transports cellular constituents from the cytoplasm to the lysosome, where the cargos are broken down by double-membrane autophagosomes [27]. Autophagy functions as a major cytoprotective process by preserving cellular homeostasis and recycling cytoplasmic constituents. However, studies suggest that autophagy is a primary form of cell death and implicates autophagic cell death in the pathological process of DOX-induced cardiomyocyte death [28]. Anthracyclines have been found to induce dysregulated autophagy, which leads to an excessive amount of cardiomyocyte death [29].

The process of cell autophagy is initiated by AMP-activated protein kinase (AMPK) activation and mammalian target of rapamycin (mTOR) inhibition, both of which have been shown to induce unc-51 like autophagy activating kinase 1 (ULK1) activation. ULK1 promotes autophagy by phosphorylation and positive regulation of the autophagy-related protein Beclin-1 (BECN1), the signaling hub in the context of autophagy [30]. Bcl-2, an early gatekeeper of autophagy control, also inhibits BECN1 [31]. Current studies on the effect of anthracyclines on autophagy have reported conflicting results. Some studies have reported that anthracyclines induce autophagy [28], while others have reported that anthracyclines inhibit autophagy [32, 33]. Similarly, studies on the effects of genetic or pharmacological suppression of autophagy have yielded mixed results, with some indicating protective effects [34, 35], while others demonstrate that sustained reduction of autophagy cannot maintain protection against DOXIC [36, 37].

Recent studies have shown that these contradictory results may be due to the different effects of DOX on autophagy at different phases. DOX initially promotes autophagy but later prevents it. Thus, DOX leads to an accumulation of autophagosomes and autolysosomes that have not yet been destroyed, increasing the damage to cardiac cells, and ultimately resulting in cell death. DOX at low concentrations induces autophagy by up-regulating the expression of BECN1 [38]. In an adult DOXIC zebrafish model, a biphasic response in autophagy was observed: activation in the early stage and suppression in the later phase that is accompanied by cardiac functional decline [39]. Moreover, overexpression of autophagy related 7 proteins, a rate-limiting autophagy regulator, leads to therapeutic effects in the late phase but deleterious effects in the early phase of adult DOXIC [39]. In a more recent study using *in vitro* rat myoblast H9c2 cell culture model, DOX was revealed to block the progression of autophagy, particularly the fusion of autophagosomes with lysosomes. This decrease in the formation of autolysosomes and contributes to the accumulation of dysfunctional mitochondria and subsequent increase in cytotoxic ROS, eventually resulting in increased myocardial cell death [33]. The transcription factor E-box binding protein (TFEB) controls lysosomal signaling and function [40, 41]. DOX inhibits the expression of TFEB in cardiomyocytes. The inhibition of TFEB leads to decreased macrophage protein expression, inhibition of autophagy flux, impairment of lysosome cathepsin B activity, and activation of cell death [42]. However, recovery and/or activation of TFEB in DOX treated cardiomyocytes prevents DOX-induced cathepsin B activity inhibition, reduces DOX-mediated ROS overproduction, weakens the activation of caspase-3, and improves the cell’s viability [42]. These studies reveal that DOX hinders the process of autophagosome destruction, resulting in the accumulation of autophagosomes and the consequent overproduction of ROS and subsequent cell death.

## 5. Ferroptosis

A recently identified intracellular iron-dependent form of cell death process called ferroptosis is distinguished by intracellular iron accumulation and lipid peroxidation [43]. Recent studies have shown that ferroptosis, a novel form of regulated cell death mediated by iron-dependent lipid peroxidation, also plays a key role in DOX-induced cardiomyocytes death.

The main contributor to cardiac damage in DOX is mitochondrial-dependent ferroptosis. By forming the DOX-${\mathrm{Fe}}^{2+}$ complex in mitochondria and inhibiting glutathione peroxidase 4 (GPX4) expression, DOX promotes excessive lipid peroxidation, resulting in mitochondria-dependent ferroptosis [44]. DOX can also insert into mitochondrial DNA (mtDNA). The damaged mtDNA accumulates to induce ferroptosis in a mtDNA quantity-dependent manner [45]. In addition, DOX reduces the expression of the heme (iron-protoporphyrin) biosynthesis rate-limiting enzyme $5^{\prime }$-aminolevulinic synthase 1 (Alas1), thereby disrupting heme synthesis, and leading to iron overload and consequent ferroptosis in cultured cardiomyocytes [45]. DOX also promotes ferroptosis in cardiomyocytes by enhancing ferritin phagocytosis by a mechanism involving nuclear receptor coactivator 4 deubiquitination *via* the spermatogenesis associated 2/CYLD (cylindromatosis) pathway [46]. The mitochondrial outer membrane protein FUNDC2 (FUN14 domain-containing protein 2) was recently reported to interact with and destabilize the mitochondrial glutathione transporter solute carrier family 25 member 11 to reduce mitochondrial glutathione levels and incite ferroptosis in DOX-treated cardiomyocytes [47]. The E3 ubiquitin ligase membrane associated ring-CH-type finger 5 (MARCHF5) is essential for preserving mitochondrial function. Both MARCHF5 and GPX4 are decreased in cardiomyocytes treated with DOX; however, maintaining MARCHF5 expression can keep GPX4 levels stable and hence prevent DOX from ferroptosis [48]. By directly interacting with sequestosome-1/p62 and ubiquitinating p62, the E3 ligase tripartite motif containing 21 (TRIM21) exerts a negative influence over redox regulation and the P62- Kelch-like ECH-associated protein 1 (KEAP1)- Nuclear factor E2-related factor 2 (Nrf2) antioxidant pathway. During oxidative stress, TRIM21-defective cells display a greater antioxidant response, eventually reducing cell death [49]. TRIM21-deficient heart tissue and cardiomyocytes show enhanced p62 isolation of KEAP1 and are protected from DOX-induced ferroptosis [50]. Acyl CoA thioesterase 1 (ACOT1) in cell lines demonstrates considerable protection against ferroptosis due to its role in the long-chain fatty acid metabolic process. Cardiomyocytes with ACOT1 knockdown are susceptible to DOX-induced ferroptosis [51]. In mouse cardiomyocytes, silencing heme oxygenase-1 (HMOX1), an essential enzyme in heme catabolism cleaving heme to form biliverdin, ameliorates DOX-induced damage, mitochondrial dysfunction, and ferroptosis *via* lowering CTGF (connective tissue growth factor) expression [52]. The importance of treating cardiac iron overload during DOX therapy is highlighted by the clinical use of the iron-chelating agent dexrazoxane, which is the only clinically approved cardiac protective agent [53].

## 6. Necroptosis

Cell necroptosis is a programmed form of necrosis mediated by DRs, or inflammatory cell death. Receptor interacting protein kinase-1 and the -3/mixed lineage kinase domain-like protein (receptor-interacting protein [RIP]1/RIP3/mixed lineage kinase domain like pseudokinase [MLKL]) pathway is activated during canonical necroptosis [54, 55]. Specific DRs, including but not limited to TNF-R1, Fas receptors, and Toll-like receptors (TLR), are also responsible for sensing a variety of stimuli that cause necroptosis [56, 57].

Recent studies have shown substantial interplay between DOX-induced necroptosis and other death modes of cardiomyocytes. DOX was shown to inhibit autophagy flux [42]. The inhibition of autophagy flux leads to RIP1-RIP3 interaction and activation of necroptosis in cardiomyocytes, which in turn leads to cell death [58]. mTOR complex 1 (mTORC1) inhibition enhances autophagy and protects cardiomyocytes against necroptosis *via* a TFEB-dependent mechanism [59]. DOX also activates necroptosis through a noncanonical RIP3/calmodulin-dependent protein kinase II (CamkII) pathway. DOX treatment increases the amount of receptor-interacting protein 3 (RIP3) in the myocardium. RIP3 binds to and phosphorylates ${\mathrm{Ca}}^{2+}$-CamkII, resulting in the opening of mitochondrial permeability transition pores and myocardial necroptosis [60]. Some drugs have been found to inhibit necroptosis in pharmacological studies, such as phosphocreatine [61], necrostatin 1 [62], Donepezil [63] and KN-93 (a CamkII inhibitor) [60]. It will be important to determine whether these agents can reduce or prevent DOXIC in cancer patients in clinical trials.

## 7. Pyroptosis

Pyroptosis is a highly inflammatory form of lytic programmed cell death [64]. The main morphological characteristics of pyroptosis include membrane perforation, cell swelling, leakage of cellular content, chromatin condensation and DNA fragmentation. Unlike apoptosis, the nucleus is mostly intact in pyroptosis. Pyroptosis has been found to be involved in many cardiovascular disorders including DOXIC [65]. The process of pyroptosis is usually incited by the formation of a large supramolecular complex termed the inflammasome as a result of various stimuli [2]. The inflammasome activates a different set of caspases from those associated with apoptosis, for example, caspase-1/4/5 in humans and caspase-11 in mice. The activated caspase-1 in turn cleaves gasdermin D (GSDMD) into the N-terminal domain. The GSDMD-N-terminal domain then forms pores in the plasma membrane, resulting in pyroptosis.

The involvement of the canonical nod-like receptor protein 3 (NLRP3) inflammasome pathway-mediated pyroptosis in the pathogenesis of DOXIC has been reported in several studies. The NLRP3/caspase-1/GSDMD axis was shown to contribute to DOXIC pathogenesis. DOX-induced dynamin-related protein 1-mediated mitochondrial fission and the ensuing NLRP3 inflammasome activation and pyroptosis could be rescued by NADPH oxidase 1 (NOX1) and NADPH oxidase 4 (NOX4) silence [66]. There is also evidence suggesting that DOX induces pyroptosis of cardiomyocytes in a GSDMD-dependent manner by directly binding to GSDMD and promoting GSDMD-N-mediated pyroptosis [67]. Similarly, DOX also causes pyroptosis of cardiomyocytes through the caspase-3/gasdermin E (GSDME) axis [68]. Human GSDME-positive SH-SY5Y and MeWo cells as well as mouse HL-1 cardiomyocytes exhibited GSDME-dependent pyroptosis upon activation of caspase-3 by chemotherapy drugs including DOX. *Gsdme*-/- mice were resistant to DOX-induced tissue damage [68]. Bcl-2/adenovirus E1B interacting protein 3 (Bnip3) is a pro-apoptotic protein. A previous study revealed the important role of Bnip3 in activating the caspase-3/GSDME axis. Cardiomyocyte pyroptosis is activated by the Bnip3/caspase-3/GSDME pathway following DOX treatment, suggesting that Bnip3 is a novel regulator of cell pyroptosis in DOXIC [69]. GSDMD is also involved in DOX-induced Bnip3-mediated mitochondrial damage and perforation in cardiomyocytes [67]. Apart from the extensively studied NLRP3 inflammasome, the nod-like receptor (NLR) family protein 1 NLRP1 inflammasome is also reported to be involved in DOX-induced pyroptosis. The E3 ubiquitin ligase TRIM25 has been reported to promote NLRP1 ubiquitination and to reduce NLRP1 stability, thereby inhibiting NLRP1-mediated pyroptosis in DOX-treated cardiomyocytes [70].

At present, some pharmacologic agents have been shown to inhibit pyroptosis in animal experiments. MCC950 (a small molecule compound) is an NLRP3 inflammasome inhibitor, which can inhibit NLRP3-mediated pyroptosis and alleviate myocardial injury induced by DOX [71]. Curcumin inhibited DOX-induced pyroptosis of cardiomyocytes in a PI3K (phosphoinositide 3-kinase)/Akt (protein kinase B pathway)/mTOR dependent manner [72]. Calycoflavone (CAL), astragalus’ primary active component, reduces the pyroptosis of cardiomyocytes attributed to DOX, by preventing the activation of the NLRP3 inflammasome [73]. By reducing the expression of NOX1/2/3, TLR2/4, and the nuclear factor kappa-light-chain-enhancer of activated B cells (NF-κB), exogenous 8-hydroxy-deoxyguanosine (8-OHdG) attenuates DOX-induced pyroptosis [74]. Ticagrelor attenuates DOX-induced pyroptosis of cardiomyocytes by targeting glycogen synthase kinase-3β/caspase-1 [75]. Nifurazine suppressed DOX-induced pyroptosis by down-regulating the protein expression of TLR4, NF-κB, thioredoxin interacting protein (TXNIP), NLRP3, caspase-1, IL-1, and the GSDMD-N terminus [76]. It will be important to determine whether these agents are effective in clinical trials to reduce or prevent DOX-induced cardiomyocyte pyroptosis in cancer patients.

Some non-coding RNAs have also been revealed to play essential roles in DOX-related pyroptosis. Terminal differentiation-induced noncoding RNA has recently been reported to direct insulin-like growth factor 2 binding protein 1 to increase the stability and expression of NLRP3, thereby inducing pyroptosis in DOX-treated cardiomyocytes [77].

## 8. Dysfunction

**Fig. 2.**
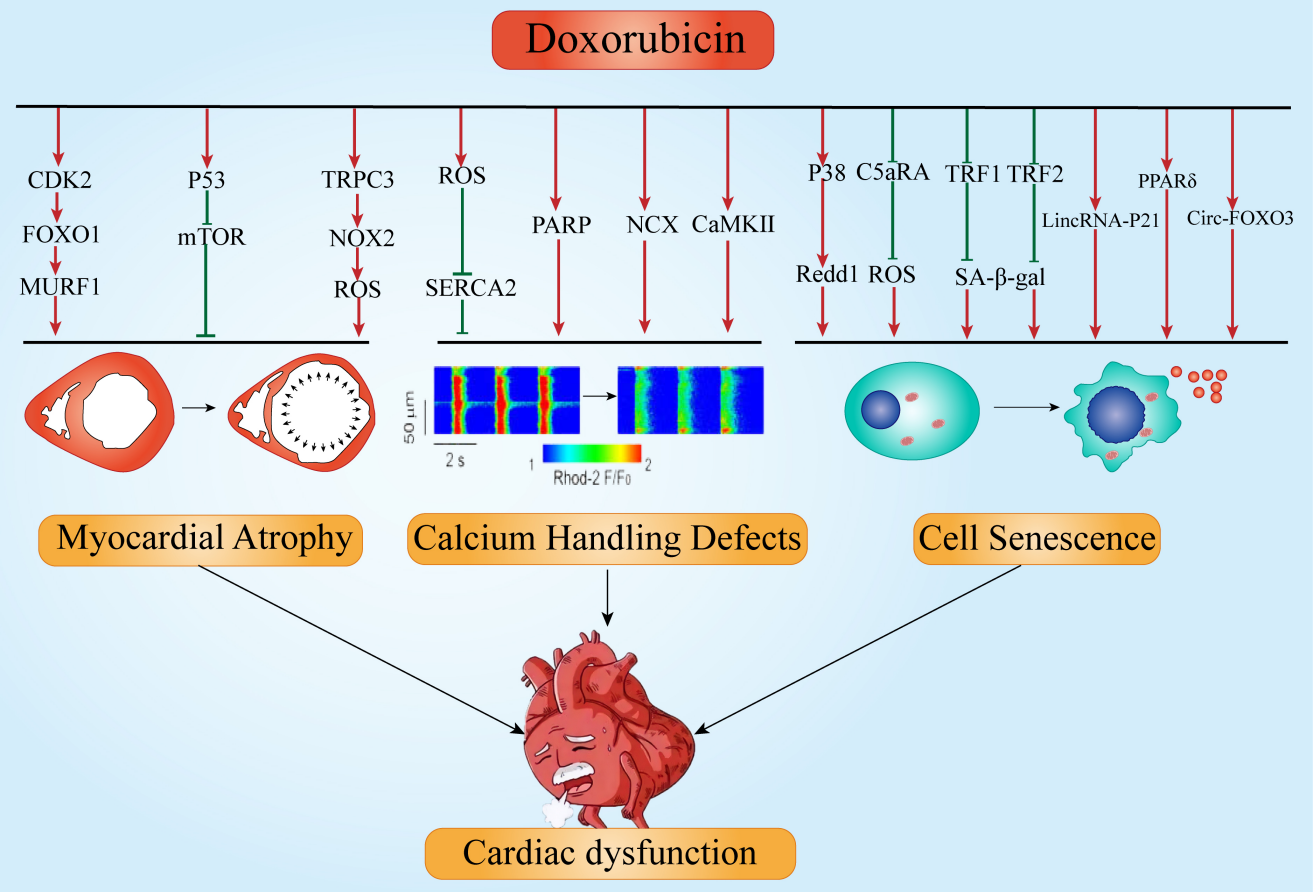
**Proposed mechanisms involved in Dox-induced cardiac dysfunction**. Dox, doxorubicin; CaMKII, calmodulin kinase II; CDK2, cyclin-dependent kinase 2; FOXO1, fork head box O1; FOXO3, fork head box O3; mTOR, mammalian target of rapamycin; MURF1, muscle RING finger 1; NOX2, NADPH oxidase 2; PARP, poly ADP ribose polymerase; PPARδ, peroxisome proliferator-activated receptor; Redd1, regulated in development and DNA damage response-1; SA-β-gal, senescence-associated β-galactosidase; SERCA2, sarcoplasm/endoplasmic reticulum ${\mathrm{Ca}}^{2+}$-ATPase 2; TRF1, telomeric repeat binding factor 1; TRF2, telomeric repeat binding factor 2; TRPC3, transient receptor potential canonical 3; ROS, reactive oxygen species; p53, tumor protein p53; p38, p38 mitogen-activated protein kinase; NCX, sodium-calcium exchanger; C5aRA, C5a receptor antagonist. Arrows indicate activation; bar-headed lines indicate inhibition.

Apart from drastic cell death, DOX also induces relatively mild cardiomyocytes dysfunction that exacerbates its cardiotoxicity. In addition to the classical manifestations of myocardial cell dysfunction caused by oxidative stress and mitochondrial dysfunction, there are also some recently focused pathological processes including myocardial atrophy, ${\mathrm{Ca}}^{2+}$ processing defects, thrombosis, cell senescence causing myocardial dysfunction (Fig. 2). We’ll focus on these pathological manifestations next.

## 9. Myocardial Atrophy

Myocardial atrophy is a significant factor in the decrease of left ventricular mass following anthracycline therapy, and cardiac atrophy is a common factor of DOX-induced cardiac dysfunction [78]. The loss of left ventricular myocardial cell mass increases with the increased level of myocardial cell damage [79]. There is evidence demonstrating that loss of myocardial mass rather than cardiomyocyte death is the major contributor to acute DOX cardiotoxicity [80]. Cardiomyocyte atrophy was viewed as an underestimated contributor to DOXIC [78].

Multiple pathways are involved in anthracycline-induced myocardial atrophy. Muscle ring finger-1 (MuRF1), a striated muscle-specific ubiquitin ligase, is required for DOX-induced cardiac atrophy in mice. MuRF1-deficient mice were protected from cardiac atrophy after DOX exposure with no loss in systolic function [81]. Fork head box O1 (FOXO1) is highly expressed in adult heart tissue and is essential for cardiac development [82, 83]. Through the transcriptional activation of atrophy-related target genes such as atrogin-1 and MuRF1, FOXO transcription factors regulate skeletal muscle atrophy in addition to apoptosis [84]. Increased FOXO1 levels in the heart result in atrophy of cardiomyocytes and loss of cardiac mass [85, 86, 87]. Depletion of FOXO1 protects against DOX-induced myocardial weight loss and decreased cardiac function [88]. NADPH oxidase 2 (NOX2) is another master regulator of myocardial atrophy. Mice lacking NOX2 display resistance to DOX-induced cardiac atrophy, which is associated with mitigated NADPH oxidase activity, oxidative/nitrosative stress, and inflammatory cell infiltration [89]. Myocardial atrophy caused by DOX is also reported to be under the control of the transient receptor potential canonical 3 (TRPC3)-NOX2 complex. The amplification of NOX2-mediated ROS signaling in cardiomyocytes can be inhibited by blocking TRPC3, thereby avoiding DOX-induced cardiac atrophy [90]. DOX is also reported to contribute to myocardial atrophy by suppressing the cell growing mTOR pathway [91]. This inhibitory effect has been demonstrated to be dependent on P53 activation, resulting in acute cardiac insufficiency and myocardial atrophy [80].

## 10. Calcium Handling Defects

DOX cardiotoxicity is associated with dysregulation of ${\mathrm{Ca}}^{2+}$ levels. Impaired calcium homeostasis is responsible for impaired cardiac contractility [4]. Subclinical cardiotoxicity of DOX is associated with disturbances in sarcoplasmic reticulum (SR)-mediated ${\mathrm{Ca}}^{2+}$ regulation [92]. Dysfunction of the SR may progress after DOX treatment, leading to late cardiotoxicity [93]. The main ${\mathrm{Ca}}^{2+}$ transporter in the SR is sarcoplasmic/endoplasmic reticulum ${\mathrm{Ca}}^{2+}$-ATPase 2 (SERCA2), which functions as a transporter carrying ${\mathrm{Ca}}^{2+}$ from the cytosol to the SR. DOX causes dysregulation of ${\mathrm{Ca}}^{2+}$ dynamics by decreasing the expression of SERCA2 [94]. Transcription factors including early growth response factor 1 and p44/42 MAPK were found to be the key factors controlling SERCA2 expression in DOX-treated cardiomyocytes [95]. DOX directly interacts with the cardiac ryanodine receptor 2 (RyR2) and SERCA2, altering their functions by attaching to proteins and interfering with SR ${\mathrm{Ca}}^{2+}$ handling by thiol oxidation [96]. DOX also promotes disturbances in cellular ${\mathrm{Ca}}^{2+}$ processing by activating poly ADP ribose polymerase (PARP) [97]. DOX was also reported to prolong the duration of action potentials by increasing ${\mathrm{Ca}}^{2+}$ influx, inhibiting ${\mathrm{Ca}}^{2+}$ release from the SR, and inhibiting sarcoplasmic ${\mathrm{Ca}}^{2+}$ extrusion through ${\mathrm{Na}}^{+}$/${\mathrm{Ca}}^{2+}$ exchange [98]. Diastolic dysfunction may be caused by impaired sequestration of intracellular free ${\mathrm{Ca}}^{2+}$ ions in DOX-treated cardiomyocytes; therefore, monitoring diastolic function is crucial for identifying early DOXIC [99]. In addition, DOX impaired intracellular ${\mathrm{Ca}}^{2+}$ mobilization and sequestration as well as the ${\mathrm{Ca}}^{2+}$transient response to β-adrenergic receptor stimulation during cardiomyocyte contractile-relaxation cycles. Angiotensin-converting enzyme inhibitors [100] and benidipine [101] ameliorated DOX-induced impairment of ${\mathrm{Ca}}^{2+}$ handling. DOX exposure-induced SR ${\mathrm{Ca}}^{2+}$ leakage was demonstrated to be CamkII-dependent, which partially resulted in impaired ${\mathrm{Ca}}^{2+}$ homeostasis. Pharmacological and genetic inhibition of ${\mathrm{Ca}}^{2+}$/CamkII weakened but did not completely eliminate the effects of DOX on ${\mathrm{Ca}}^{2+}$ dysregulation [102]. Further studies revealed that RyR2 phosphorylation at CamkII (pS2814) increased following 2 weeks of DOX treatment in a mouse model, coinciding with the occurrence of arrhythmic ${\mathrm{Ca}}^{2+}$ waves [103]. DOX-impaired SR-${\mathrm{Ca}}^{2+}$ uptake was also associated with lesions in the electron transport system of skeletal muscle mitochondria [104]. DOX exposure incited pronounced changes in electrical activity of cardiomyocytes, which was associated with structural T-tubule disturbances [105].

Preventing ${\mathrm{Ca}}^{2+}$ processing disorders caused by DOX is also an important part of DOXIC treatment. L-carnitine plays a cardioprotective role by preventing DOX-induced diastolic ${\mathrm{Ca}}^{2+}$ overload [106]. Renozine can normalize ${\mathrm{Ca}}^{2+}$ and ${\mathrm{Na}}^{+}$ processing in the heart, thereby improving DOX-induced diastolic dysfunction [107]. Salvianolic acid B was reported to relieve ${\mathrm{Ca}}^{2+}$ overload in cardiomyocytes by inhibiting TRPC3 and TRPC6 [108]. Similarly, in rats with DOX-induced cardiomyopathy, the use of a left ventricular assist device (LVAD) restored SR ${\mathrm{Ca}}^{2+}$ ATPase activity and subsequently improved ${\mathrm{Ca}}^{2+}$ handling and contractility [109]. Our previous study also demonstrated the favorable role of circular RNA itchy E3 ubiquitin protein ligase (CircITCH) in improving DOX-induced ${\mathrm{Ca}}^{2+}$ handling defects by derepressing the inhibitory effects of miR-330-5p on SERCA2a, thereby restoring SERCA2a expression and relieving DOX-induced contractile dysfunction [110].

## 11. Thrombosis

The second leading cause of death in cancer patients is thromboembolic disease [111, 112]. Chemotherapy agents associated with DOX are known to cause thrombotic complications in cancer patients [113].

DOX promotes thrombosis through a variety of pathways. DOX has a direct effect on platelets. DOX-induced venous thrombosis is strongly dependent on platelet activation. Aspirin and clopidogrel, the two most potent antiplatelet medications, have demonstrated considerable antithrombotic activity in DOX-exacerbated stasis-induced thrombosis [114]. The procoagulant activity of platelets increased by DOX can be related to the increased platelet exposure to phosphatidylserine (PS) and PS-bearing microparticles, in which intracellular ${\mathrm{Ca}}^{2+}$ increase and ATP depletion play important roles [115]. Recent studies have shown that DOX induces the release of P-selectin expressed in platelet α particles to promote platelet activation [116].

DOX-induced vascular toxicity also contributes to DOX-induced venous thrombosis. DOX administration was found to be correlated with smaller blood vessels (<15 µm) and rapid vasoconstriction (2–5 min) in mice [117]. DOX-induced acute vasotoxicity involves increased platelet adhesion to endothelial cells, leading to microthrombus formation and impaired blood flow [118]. In addition, DOX also affects vascular endothelial cells. Specifically, DOX modifies the hemostasis equilibrium of endothelial cells by suppressing the endothelium-based protein C anticoagulant pathway. Zymogen protein C (ZPC) is the precursor to activated protein C (APC), a pivotal endogenous anticoagulant in human blood. Endothelial protein C receptor (EPCR) and thrombomodulin are two types of endothelial cell surface receptors required for the transformation from ZPC to APC. When human umbilical vein endothelial cells (HUVECs) are treated with DOX, the quantity of thrombomodulin on the cell surface increases and the level of EPCR decreases. As a result, HUVEC’s capacity to transform protein C into activated protein C declines while its procoagulant activity increases [113].

## 12. Cell Senescence

Cardiomyocyte senescence is assumed to be one of the main contributing factors to the onset of DOXIC [119, 120]. Cardiomyocytes treated with DOX have a senile-like phenotype and exhibit aberrant troponin phosphorylation patterns, which may cause ineffective ventricular contraction [121]. Anthracycline-induced senescent cell accumulation is associated with long-term deterioration of cardiovascular homeostasis [3]. To date, most reports have demonstrated the pro-aging effects of anthracyclines on various types of cardiac cells.

DOX induces the increase of senescence phenotype as evidenced by increased P16INK4a (official gene symbol *CDKN2A*, cyclin dependent kinase inhibitor 2A) expression in cardiomyocytes [120]. Furthermore, studies have shown that different DOX doses directly lead to different outcomes of cardiomyocytes, with low dose inducing cardiomyocyte senescence while high dose inducing apoptosis [122]. While low-dose DOX can cause downregulation of both telomeric repeat binding factor 2 (TRF2) and telomeric repeat binding factor 1 (TRF1), increased β-galactosidase activity, and induction of cardiomyocyte senescence, high-dose DOX can significantly reduce the expression of TRF1 and overtly increase the expression of TRF2, favoring cell apoptosis [122]. Peroxisome proliferator-activated receptor δ (PPARδ) is a ligand-activated transcription factor that controls lipid metabolism, insulin sensitivity, and inflammation. Low doses of DOX promote the expression of PPARδ and enhance PPARδ-mediated sequestration of Bcl 6 to prevent the anti-senescence effects of Bcl 6, thereby exacerbating senescence [123]. As a downstream effector of p38 MAPK, regulated in development and DNA damage response-1 (Redd1) promotes DOX-induced cardiomyocyte senescence by promoting NF-κB signaling pathway through p65 phosphorylation and the nuclear translocation of NF-κB, while Redd1 knockdown dramatically decreases cardiomyocyte senescence [124]. Cathepsin K blocks DOX induced translocation of apoptosis inducing factor from the mitochondria to the nucleus, thereby inhibiting DOX-induced premature aging in cardiomyocytes [125]. Plasminogen activator inhibitor-1 is an efficient serine protease inhibitor that can prevent DOX-induced cell senescence by lowering the generation of reactive oxygen species and increasing catalase and other antioxidants [126]. Long intergenic non-coding RNA p21 (lincRNA-p21) is implicated in DOX-related cardiac senescence. Silencing lincRNA-p21 effectively prevents DOX cardiotoxicity by regulating the Wnt/β-catenin signaling pathway and reducing oxidative stress [127]. Sirtuin (SIRT6) is a key anti-aging molecule that can regulate various cellular processes related to aging. Overexpression of SIRT6 protects cardiomyocytes from DOX-induced senescence [128]. Fork head box O3 (FOXO3) circular RNA has been proven to be a contributor of DOX-induced cardiac senescence. Mechanistic studies have found that FOXO3 circular RNA interacts with the anti-aging protein ID-1, E2F transcription factor 1 (E2F1), anti-stress focal adhesion kinase, and hypoxia-inducible factor 1 (HIF1) to promote aging [129]. Parkin promotes mitochondrial autophagy to retard cardiac aging by promoting K1 linked polyubiquitination of TBK63 (K63-polyubiquitination of TANK-binding kinase 1) [130]. The tumor suppressor gene programmed cell death 5 (PDCD5) has been shown to promote cardiomyocyte senescence and apoptosis by decreasing Parkin-mediated mitophagy, while PDCD5 deficiency can attenuate natural cardiac aging and DOX-induced premature cardiac aging [131].

In addition to cardiomyocyte senescence, DOX also induces the senescence of other types of cardiac cells. DOX induces a DNA damage response in human cardiac progenitor cells, which results in telomere shortening and senescence [132]. Transient DOX treatment contributes to the onset of long-term senescence associated with a reduction in vascular endothelial growth factor receptor 2 levels in endothelial cells [133]. DOX-induced senescence has also been reported in human endothelial progenitor cells and vascular smooth muscle cells [134, 135].

There have been many studies on pharmacologic agents to retard cardiac senescence induced by anthracyclines. Experimental evidence suggests that anthracycline-induced cardiomyocyte senescence is eliminated by testosterone, and that testosterone preconditioning prevents DOX-induced downregulation of TRF2 and activation of p53 in H9C2 and neonatal mouse cardiomyocytes, resulting in decreased expression of the aging markers senescence-associated β-galactosidase [SA-β gal] and p16INK4a [136]. Honokiol effectively protects cardiomyocytes from DOX-stimulated aging. This protective effect is achieved through inhibition of TXNIP (thioredoxin interacting protein)-activated NLRP3 inflammasome [137]. Folic acid delays the senescence of cardiomyocytes, the effect of which may be mediated *via* mitigated ER stress responses [138]. Pummelo delays the onset of DOX-induced cellular senescence by lowering intracellular oxidative stress, maintaining the availability of glutathione (GSH), and increasing GSH enzyme activity and expression [139]. Roflumilast reduces DOX-induced cardiomyocyte inflammation and cell senescence by upregulation of SIRT1 [140]. SIRT1 activators may be used clinically to prevent DOX-induced cardiotoxicity by safeguarding progenitor cells [141]. Resolvin E1 (RvE1), an endogenous pro-resolving mediator, has been shown to reduce endothelial senescence induced by DOX [142]. Pharmacological senolysis using Navitoclax reduces DOXIC and improves cardiac function in mice [143]. It will be important to determine whether these anti-senescence agents are effective in clinical trials to reduce or prevent DOXIC in cancer patients.

## 13. Conclusions and Perspectives

DOX results in cardiotoxicity, due to multiple forms of cell death and myocardial dysfunction. DOXIC is an important public health problem. Though some clinical trials have shown that non-drug interventions such as exercise, healthy lifestyle, control of risk factors and treatment of comorbidities, as well as pharmacologic interventions including β blockers, angiotensin converting enzyme inhibitors, angiotensin receptor antagonists, statins, dexrazoxane and liposome preparations [10, 11, 12], have mitigated the effects of DOX, we are far from achieving a complete solution to this important problem. Hence, it is important to have a thorough understanding of its toxicity and mechanisms of action, in order to further develop novel therapeutics to avoid or mitigate the effects of DOX. This mini-review highlights recent advances in the mechanisms of cell death and dysfunction in DOX cardiotoxicity and the therapeutics targeting these mechanisms. In addition to cardiotoxicity, vasotoxicity and arrhythmias associated with DOX are currently the subject of research studies. Finally, considering the ever-increasing survival rates in patients with cancer and the aging population, how cardiovascular toxicities of anthracycline are properly managed in these patients will become crucial. Further studies on the mechanisms concerning the cardiotoxicity of anthracyclines will help to mitigate the cardiotoxicity of anthracyclines, thus holding the promise of improving the life quality and preventing cardiovascular death associated with chemotherapy in cancer patients.
